# Growing old together: What we know about the influence of diet and exercise on the aging host's gut microbiome

**DOI:** 10.3389/fspor.2023.1168731

**Published:** 2023-04-17

**Authors:** Chequita N. Brooks, Madeline E. Wight, Oluwatobi E. Azeez, Rachel M. Bleich, Kevin A. Zwetsloot

**Affiliations:** ^1^ Department of Biology, Appalachian State University, Boone, NC, United States; ^2^ Department of Public Health and Exercise Science, Appalachian State University, Boone, NC, United States

**Keywords:** microbiota, gastrointestinal tract, dysbiosis, age, physical activity, inflammation, inflammaging

## Abstract

The immune system is critical in defending against infection from pathogenic microorganisms. Individuals with weakened immune systems, such as the elderly, are more susceptible to infections and developing autoimmune and inflammatory diseases. The gut microbiome contains a plethora of bacteria and other microorganisms, which collectively plays a significant role in immune function and homeostasis. Gut microbiota are considered to be highly influential on host health and immune function. Therefore, dysbiosis of the microbiota could be a major contributor to the elevated incidence of multiple age-related pathologies. While there seems to be a general consensus that the composition of gut microbiota changes with age, very little is known about how diet and exercise might influence the aging microbiome. Here, we examine the current state of the literature regarding alterations to the gut microbiome as hosts age, drawing particular attention to the knowledge gaps in addressing how diet and exercise influence the aging microbiome. Further, we will demonstrate the need for more controlled studies to investigate the roles that diet and exercise play driving the composition, diversity, and function of the microbiome in an aging population.

## Introduction

Aging is a gradual and irreversible physiological process that occurs in all biological life forms. The aging phenotype is characterized by genomic instability, telomere attrition, epigenetic alterations, loss of protein homeostasis (proteostasis), deregulated nutrient sensing, mitochondrial dysfunction, cellular senescence, stem cell exhaustion, and altered intercellular communication in multicellular eukaryotic organisms (1). This definition has been recently updated to include a focus on the dysbiosis of host microbiota (2). The dysregulation of these physiological processes results in the development of pathological conditions, such as cardiovascular disease, metabolic disease, neurodegenerative disease, musculoskeletal disease, and immune system diseases (3). One factor common amidst all of these chronic conditions is inflammation.

The immune system is key to host defense against pathogenic organisms and protecting against disease. Immunosenescence—age-related remodeling of the immune system—leads to a decline in the protective components of the immune system and chronic, low-grade inflammation, termed “inflammaging” (4). Aged individuals are more susceptible to infectious diseases and have a higher risk of developing the noncommunicable diseases described above; therefore, immunosenescence is a major contributor to the declining health of the aging population. The gut microbiome is considered to be an important factor contributing to host health, as it plays a significant role in immune system function (5). Arguably, two of the most influential lifestyle behaviors that promote health are consuming a nutritious, well-balanced diet and partaking in regular physical activity. Diet and exercise are well-known to modulate immune function and inflammation (6); however, less understood is the interaction between diet, exercise, and the gut microbiome with aging. This is further exacerbated as the interactions between diet, exercise, and the gut microbiome have not been thoroughly studied in the context of age. This review will discuss how the gut microbiome changes with age, the role of the gut microbiome in immune function and inflammation, and how diet and exercise have the potential to influence the gut microbiome to promote health in the aging population. Lastly, we stress the need for well-controlled investigations to study the complex interactions between the host gut microbiota, diet, and exercise in the aging population.

## Role of the gut microbiome in health and disease

The immune system contains barrier, recognition, elimination, and memory functions, along with multiple cell types, and chemical mediators to derive highly-sophisticated immune responses. The immune system can then protect the host from pathogenic organisms, as well as regulate the balance between pro- and anti-inflammatory states. Individuals with weakened immune systems are more susceptible to infections, chronic, low-grade inflammation, and developing non-communicable diseases (5). The human gastrointestinal (GI) tract plays a vital role in the immune response as it contains a very complex and diverse population of microorganisms, i.e., the gut microbiota, that can influence health and disease of the host. The gut microbiome is an integral factor in determining the host immune response and dysregulation of the gut microbiota may be responsible, in part, for chronic diseases involving the immune system (7).

The gut microbiome encodes over 3 billion genes to produce a multitude of metabolites, whereas the human genome has been characterized as encoding only ∼23,000 genes. This highlights the magnitude of the “superorganism” known as the gut microbiome and its symbiosis with the host (8). Many of the over 2000 human-associated microbial species identified can be classified into 12 different phyla, ∼93% belonged to Proteobacteria, Firmicutes, Actinobacteria, and Bacteroidetes (9, 10). Confounding literature within the field has made it difficult to agree on the functions of some phyla and their influence on host health (11). Gut microbiota vary taxonomically and functionally, not only between individual hosts, but also in different parts of the GI tract within the same individual host (12). The gut microbiome is less taxonomically diverse than some other human microbial communities, such as skin, and has a high degree of functional redundancy (12, 13). Moreover, the richness and diversity of the microbiome can also be impacted by age, environmental and behavioral factors, such as diet and exercise (8). Microbiota richness and diversity increase drastically from birth to age 3 and fluctuates until reaching adulthood, when composition becomes stable and dominated by the three bacterial phyla Firmicutes, Bacteroidetes, and Actinobacteria (8).

The gut microbial community can be influenced by the availability of nutrients in the GI tract, such as dietary fiber (14) and contributes to optimal digestion and absorption of nutrients. It is responsible for the production of metabolites essential to host health, including vitamins and short-chain fatty acids (SCFAs) (15–17). SCFAs are highly valuable to the host, as they provide essential substrates for intestinal cells, can improve the gut barrier integrity, and regulate the immune system and inflammatory responses (18). SCFAs result from saccharolytic fermentation of carbohydrates, such as inulin and plant cell wall polysaccharides, which otherwise would escape digestion and absorption from the small intestine (18). The predominant SCFAs are acetate, butyrate, and propionate and are found at a proportion of 3:1:1 in the GI tract where they are involved in regulating a multitude of cellular processes (19). Butyrate is the major energy source of colonocytes (20) and also increases gut barrier integrity (21). SCFAs may be one of the most important gut microbial products affecting a variety of host physiological processes, including energy utilization and host-microbe signaling. A healthy intake of dietary fiber will stimulate fermentation of complex carbohydrates and the production of SCFAs, bolstering the defense of the intestinal mucosal barrier and inhibiting pro-inflammatory signals in the gut (22). Further, SCFAs may act as pleiotropic immunomodulators and reduce the severity of inflammatory diseases (23).

Due to immense microbial heterogeneity, it is difficult to clearly define the “optimal” microbiota to promote host health; however, the richer and more diverse a microbial community, the better it can function to ward off foreign pathogens, bolster host immune function, maintain the structural integrity of the gut mucosal barrier, and produce beneficial metabolites (8). The diversity of the microbiome allows for a wide range of functions, including the production of metabolites that can influence multiple physiological processes in the host. Conversely, a microbiome that lacks richness and diversity is suggestive of dysbiosis, as it may no longer perform functions to promote host health (24).

## The aging host

Although lifespan has dramatically increased over the past century due to significant advancements in research, medicine, and public health, healthspan has not. In other words, the number of relative years spent free of disease or chronic debilitating conditions as we age has remained fairly constant (25). Therefore, the scientific and medical communities are faced with the urgent challenge of investigating the underlying mechanisms that cause age-related health disorders and develop feasible preventions and treatments for the aging population. Inflammaging likely contributes to a variety of age-related conditions including atherosclerosis, heart failure, chronic obstructive pulmonary disease, type 2 diabetes-mellitus, sarcopenia, osteoarthritis, osteoporosis, Parkinson's disease, and Alzheimer's disease (26). The presence of inflammaging leads to immune dysfunction and suppression of immune cells, including regulatory T and B lymphocytes and macrophages (27, 28), which can result in an increased risk of infection and poor response to vaccines (29).

Studies performed on long-lived people (octogenarians through centenarians) suggest that healthy aged individuals possess the ability the manage inflammaging with anti-inflammatory responses (30, 31). The gut microbiome may play a large role in this response, as the host microbiota interacts with a multitude of host physiological systems to promote normal function and health. While a healthy gut microbiome can help to mitigate chronic inflammation, dysbiosis may exacerbate chronic inflammation (8). During aging, the intestine undergoes a loss of integrity, which enables the translocation of gut microbiota into other bodily tissues, inducing an inflammatory response from immune cells, such as neutrophils and Th17 lymphocytes (32). Germ-free mice co-housed with normal old mice (18–22 months) displayed increased pro-inflammatory cytokine production, whereas germ-free mice co-housed with normal young mice (2–4 months) did not (33). The resulting shift in the inflammatory state of the germ-free mice is likely due to microbial transplant from the old mice to the germ-free mice (*via* consumption of mouse droppings; “coprophagy”), thus establishing a gut microbiome that reflects that of the co-housed mice. The increase in systemic pro-inflammatory signals is likely due to the microbiome composition of the aging host, as these microbiomes are more susceptible to the invasion of pathogens, compared to a young microbiome. These results also demonstrate that the inflammatory state was not derived from the host.

Further, the loss of intestinal integrity results in metabolic endotoxemia, or the increase of lipopolysaccharides in the blood following the ingestion of fat-rich meals, which also exacerbates chronic inflammation (34). However, the concentration of lipopolysaccharides in the blood has been shown to decrease with the ingestion of probiotics containing inulin (61.5%), lactitol (34.6%), and aloe vera gel (3.9%) (35). This result is likely due to the action of the microbiome, as inulin is a non-digestible carbohydrate that stimulates the activity of *Bifidobacteria* and *Lactobacilli* (36). It is clear from this example that the host diet can influence not only microbiome composition, but how the microorganisms interact directly and indirectly with the host system.

## The aging microbiome

The key to understanding how external factors such as diet and exercise might influence the gut microbiome is building an understanding of the changes to the microbiome that occur as part of the aging process. From birth, the gastrointestinal tract begins to be colonized with bacteria to form the gut microbiome. A person's gut microbial diversity increases with age and exposure to changing environments, for example stress, diet, and exercise. This emphasizes the sensitive nature of microflora population fluctuations. There are three pivotal stages in the development of the gut microbiome: birth, adolescence, and old-age (15). Substantial changes occur in human gut microbiota during the first two years of life, as the gut microbiota of neonates shifts during development to be more similar to adult microbiota by 24 months of age (37) and is continuing to increase in communal diversity by five years of age (38). Gut microbiome diversity appears to be relatively constant from adolescence through adulthood; however, alterations in the microbiome have been observed between the 60–80 year-old- and >80 year-old-individuals (32). Typically, a “resilient” microbial community, one which is unlikely to be invaded by novel taxa, is considered one with the greatest diversity (39, 40). In the context of the gut microbiome, this has often led researchers to define “healthy” microbiomes as those that are the most diverse (41, 42). Furthermore, many previous investigations place importance on the ratio of Firmicutes to Bacteroidetes for health status since these phyla constitute large proportions of bacterial content in the gut microbiome (43); however, conflicting findings from the many cross-sectional studies with aged populations make it difficult to determine the functional significance of the Firmicutes:Bacteroidetes ratio in the context of aging (44–47).

In Japanese subjects, gut microbiome diversity increased between ages 0–10 years, plateaued from 10 to 50 years, increased from 50 to 80 years, and then decreased from 80 to 100. Interestingly, there was a positive correlation between age (0–100 years) and the genera *Bacteroides* and *Eubacterium*, as well as a negative correlation with age to the genus *Bifidobacterium* (48). In adults from the Western hemisphere, the most abundant organisms found in fecal samples are known fiber degraders, including *Eubacterium* spp., *Roseburia* spp., and *Subdoligranulum variabile* from the phylum Firmicutes (49). In the case of *Bifidobacterium*, there is evidence that an increase in ingestion (e.g., probiotics) can bolster immune tolerance (50) and help with age-related maladies such as constipation (51). *Eubacterium* abundance was found to be correlated with host immune response in cross-sectional cohorts aged 20–40, 60–80, and 100+, while *Eubacterium limosum* content was found to increase in centenarians (44). *Eubacterium limosum* has previously been found to produce butyrate, acetate, and propionate, which decreased IL-6 production in T84 cell cultures (52). A chow diet supplemented with *Eubacterium limosum* also decreased colonic damage and shrinkage from dextran sulfate sodium-induced colitis in BALB/C mice (52). These mouse results likely translate well to the human model, as *Eubacterium* are one of the few genera where the average relative abundance is incredibly similar between mouse and human guts (53). These results indicate that the long-life phenotype in hosts may be connected to not just bulk diversity of the gut microbiome, but also to key functions those taxon perform. A limitation common to many cross-sectional studies in the literature is being able to determine whether the changes in microbiome diversity observed in elderly cohorts is a benefit or detriment to host health. To the best of our knowledge, longitudinal studies on the microbiome with aging do not exist, emphasizing a major limitation of the current literature. Therefore, there is a great need for controlled, longitudinal studies that track the progression of alterations to the gut microbiome with aging.

While a focus on microbial presence, absence, and relative abundance has helped to characterize the periods of change in gut microbiomes with aging, it may also serve investigators well to focus on bacterial functions carried out in the aging host. Studies have revealed that transcriptomic profiles are much more variable across individuals, whereas genomic profiles can remain relatively constant (54, 55). Therefore, multi-omic approaches that characterize not only the metagenomic (microbiome content), but also the metatranscriptomic (gene expression), metaproteomic (protein content), and metabolomic (metabolic products) profiles, provide a far more comprehensive assessment of the diversity and function of the microbiome, compared to any single analysis alone (56).

## Influence of diet on the aging microbiome

Diet is considered one of the most influential factors for the establishment of the gut microbiome. Regional communities with distinct diets can have very different gut microbial communities. Immigration to the United States from non-western nations has been associated with a loss of overall microbial diversity, a loss of *Prevotella* strains, a decrease of fiber-degrading enzymes, and an increase in *Bacteriodes* (40). Dietary changes can also drive rapid alteration of the composition of the gut microbiota (57). Human diets are composed of macronutrients, which provide energy and micronutrients, aiding with essential functions in the body. Adequate nutrition is necessary for proper cellular function, including immune cells, with certain micronutrients like vitamins and zinc having specific roles in maintaining the immune system and reducing inflammation (16). Chronic low-grade inflammation and malnutrition can present long-term health issues, such as leaky gut syndrome (58). Maintaining gut microbiome homeostasis promotes healthy intestinal permeability and preventing further bacterial and nutrient loss (59). Conversely, additions to the diet from compounds like dietary emulsifiers have been demonstrated to alter the composition of the microbiota which, in turn, induced low-grade inflammation (60).

Many metabolic processes occur throughout the GI tract, where nutrients are digested and metabolites absorbed across the intestinal mucosa into the bloodstream (61). Several steps in digestion and absorption rely on a properly functioning gut microbiome. There is irrefutable evidence that different diets across the globe, particularly those varying in macronutrient and fiber composition, dramatically impact health and disease, due in part though alterations in the composition and function of the gut microbiome (62). Healthy, well-balanced diets, rich in fiber, as well as calorically-restricted diets [combined with ≥20 grams of fiber per day (63, 64)] can extend longevity; while a high-fat, low-fiber diet can decrease longevity (65). It is recommended that adults consume 25 and 38 grams of fiber per day for women and men, respectively (66); however, Westernized diets that include processed foods high in fat, sugar, and salt [containing only ∼15 g of fiber per day (67)] can lead to increased endotoxin-producing bacteria that can potentially trigger the immune system through pathogen-associated molecular patterns (68). On the contrary, Mediterranean diets, those associated with consumption of nuts, oily fish, fruits, vegetables, cereals, and red wine [typically containing ≥30 grams of fiber per day (69)], were linked to higher abundance of fiber-fermenting SCFA-producing bacteria, like *Roseburia* and *Faecalibacterium* (70). Furthermore, observing long-term dietary patterns, Bolte et al. (2021) reported that regular consumption of legumes, breads, fish and nuts was associated with lower abundance of opportunistic bacteria that have been linked to inflammation and reduced inflammatory markers overall in the stool (70).

Aging typically alters diet and nutrient intake due to changes in appetite, food sensitivities, or access to proper nutrition. The aging host's diet is determined by a variety of factors, including living situation, independence, energy expenditure, a decline in masticatory function, and loss of smell (71). In old age, the host may also experience a slowing in digestion time and appetite decreases in a process known as physiologic hunger (72, 73). This includes a significantly lower energy intake and lower consumption of micronutrients in elderly people, compared to younger adult populations (74). Vitamins A, E, and D intake have all been reported to be below the recommended value in the elderly, and are major contributors to anti-inflammatory responses and inflammation regulation (74). Studies also increasingly demonstrate a correlation between reduced protein intake and aging (8, 15). In a 6-year study on protein intake in elderly Danish populations, lean body mass and muscle retention was found to be higher in individuals who consumed more of the dietary amino acid leucine than in populations who consumed less protein (75). Further, protein supplementation, combined with controlled physical activity, resulted in increased fat-free mass and strength and lower inflammatory markers in elderly individuals (76). These studies highlight the seen and unseen influences of nutrient intake on the aging population.

These shifts in diet often trend toward a sub-optimal microbiome, leading to dysbiosis in aged individuals. This includes a decrease in diversity and butyrate-producing microbes, with an increase in facultative anaerobes, like *Proteobacteria*, that tend to favor a more inflammatory state (50). There is also an altered ratio of Firmicutes to Bacteroidetes as Firmicutes populations decline with aging (77). Elderly individuals that live in long-term care facilities have a reduced microbial diversity, compared to community-dwellers, which is related to dietary changes such as alterations in consumption of vegetables, fruits, and meat (78). A few studies have examined different nutritional interventions to improve gut microbiota health in elderly individuals, but more are needed to target this population with their specific nutritional needs. Some probiotics have promoted *Bifidobacteria* growth, while decreasing opportunistic pathogens in the elderly (77). Use of prebiotic galacto-oligosaccharides have shown efficacy in restoring the gut microbiota, including increasing *Bifidobacteria* and *Lactobacilli*, and reducing inflammation (79). On the contrary, there is conflicting evidence that other probiotic supplementation did not significantly improve inflammation or infections in elderly patients (80). Future studies are needed to examine the impact of such supplementation on the composition of gut microbiota.

There is a great need for well-controlled, longitudinal studies examining diet-induced changes to the gut microbiota, especially with age. Further analysis of how changes to the diet can influence the diversity, composition, and function of the gut microbiota will be critical moving forward. Additionally, controlled studies for determining how dietary interventions may alter the microbiota, and if the efficacy of these treatments change with age, will be essential for improving health in aging populations.

## Influence of physical activity/exercise on the aging microbiome

Regular physical activity/exercise not only increases cardiorespiratory fitness, but also promotes health in a dose-dependent manner by reducing all-cause mortality and the risk of developing or progressing chronic diseases, such as cardiovascular disease, certain cancers, and metabolic disease (81, 82). Moreover, inflammaging and immunosenescence pose significant health risks that determine morbidity and mortality in the elderly especially since most, if not all age-related diseases have an underlying pathogenesis involving inflammation (83). Regular physical activity/exercise can reduce chronic, low-grade inflammation and enhance immune function (84, 85), but our lack of understanding on the impacts of exercise throughout the lifespan leaves much to be desired in terms of investigating the underlying mechanisms for how exercise provides health benefits (86). Autophagy, the process by which cells clean out and degrade cellular debris to maintain normal physiological function (87), is enhanced by lifelong physical activity/long-term exercise training (88, 89); therefore, providing more evidence to the importance of physical activity/exercise on promoting health and longevity. Furthermore, while the gut microbiome plays a significant role in inflammation and host immune function to promote health (7), it is less clear how exercise influences the gut microbiome, particularly with aging.

Findings from both animal and human studies suggest that exercise training alters the composition and diversity of host gut microbiomes, although it has been difficult to determine the exact mechanisms and degree to which exercise alters the gut microbiome. This is due to inconsistencies controlling for diet, age, and species, as well as the duration, intensity, and mode of the exercise intervention. This lack of control has, in turn, led to contradictory results among the limited number of studies available in the literature. For example, rodent studies have reported that exercise increases the Firmicutes:Bacteroidetes ratio (90–92), while others report changes in the opposite direction (93–96), or no change at all (97–99). In one of the more well-controlled cross-sectional animal studies, Allen et al. (2015) reported young (6-wk-old) mice that performed six weeks of voluntary wheel running (VWR) displayed lower gut microbiome richness than either forced treadmill running (FTR) or sedentary mice over the same time. Further, the overall abundance of the phyla Bacteroidetes and Firmicutes was not altered by treatment, but Tenericutes and Proteobacteria significantly increased in FTR mice, compared to sedentary and VWR (100). Bacteria of these phyla present the endotoxin lipopolysaccharide to gut epithelial and immune cells, which could implicate a GI tract pathology in the FTR mice (101). Within the phylum Firmicutes, the genus *Dorea* increased in the FTR mice, compared to sedentary (100). A high abundance of *Dorea spp.* has been associated with negative health outcomes (102); however, no work has been done to resolve the functions associated with *Dorea* spp (103). A major take away from this study is that the type of exercise stimulus (VWR vs. FTR) may alter gut microbial communities differently, such that FTR is a more stressful stimulus for mice and may be detrimental to gut microbiota homeostasis, compared to VWR.

Additionally, when sedentary germ-free mice receive microbial transplants from mice that previously performed 6 weeks of VWR, exercise-induced alterations in the gut microbiome persist and provide beneficial effects to the recipient host, such as bacterial community colonization, reduced inflammatory responses, gut-derived SCFAs, and protection against experimentally-induced colitis (104). Further, exercise recipient germ-free mice display higher butyrate to acetate ratios and butyryl CoA:acetate CoA transferase enzyme expression, compared to control recipient germ-free mice; suggesting that functional properties of the newly colonized gut microbiome persist as well to promote gut homeostasis in the recipient host (104). Similarly, Matsumoto et al. (2008) reported that young (6-wk-old) male rats that performed 5 weeks of VWR significantly increased butyrate:acetate ratio in the cecum (97). Findings from animal studies illustrate the need to better contextualize changes in microbial community species with regards to functional characteristics, rather than just richness and diversity properties since seemingly minor changes to the microbial community can produce significant alterations in the GI tract.

While human studies, by nature, are far more difficult to control for all potential confounding variables, the existing literature investigating the influence of exercise on the gut microbiome in humans is even less clear than animal studies reviewed above—of these, cross-sectional studies are predominant. Clarke et al. (2014) observed differences in gut microbiota composition and diversity in professional rugby athletes, compared to sedentary controls of similar body mass indexes (BMI). Athletes displayed significantly higher relative abundances of more than 40 taxa—specifically in the phyla Firmicutes; and lower abundance of only 3 taxa—specifically in the genera *Lactobacillaceae, Bacteroides*, and *Lactobacillus* (105). Barton et al. (2018) extended these findings to demonstrate that the functional capacity of the athlete's microbiota was significantly enhanced, compared to controls, such that metabolic byproducts of amino acids and carbohydrates, along with SCFA concentrations were elevated (106). The few other studies in athletic populations have demonstrated varied results, with some reporting similar changes to the gut microbiome as above (107, 108), while other report no differences (109, 110). From athletes to normal healthy young adults, it has been reported that cardiorespiratory fitness level is highly correlated with the composition and diversity of the gut microbial community. In fact, both Estaki et al. (2016) and Durk et al. (2018) reported that, even after accounting for other confounding variables (such as diet and body composition), VO_2peak_ accounted for ∼20% of the variation in taxonomic diversity (e.g., Firmicutes to Bacteroidetes ratio) between participants with varied fitness levels (111, 112). Further, VO_2peak_ directly correlated with metagenomic functions of participants' microbiota, with associations to genes related to SCFA biosynthesis (specifically butyrate), bacterial chemotaxis, and motility (112). Despite the quality of the aforementioned studies, they are all limited by their cross-sectional design and fail to account for variability between individuals and treatment groups.

Mixed results have been reported in the limited number of longitudinal studies that have used an exercise training intervention in humans. Allen et al. (2018) conducted a study on young, sedentary individuals with a 6-week endurance exercise (EE) regimen, followed by 6 weeks of detraining. Lean individuals displayed increased abundance of the genera *Faecalibacterium* and *Lachnospira* and a decrease in *Bacteroides* with EE. These results suggest that microbial composition and gut-derived SCFAs were altered by exercise training, but returned to baseline levels within 6 weeks; however, changes were dependent on BMI at the start of the study (113). Conversely, only modest shifts in microbial taxonomy and function were observed in overweight women in response to a 6-week EE regimen, with the abundance of several taxa appearing to differ slightly with exercise, compared to baseline (114). Cronin et al. (2018) utilized a combination EE and resistance exercise (RE) for 8 weeks in healthy adults, but observed no changes in microbiome diversity and only subtle changes in microbial community composition and function (115). When comparing EE vs. RE exercise modalities in young, healthy adults over an 8-week regimen, Bycura et al. (2021) report substantial changes in gut microbiome composition occur in the first few weeks of EE, but then regress back to pre-exercise levels by 5 weeks into the intervention, whereas no changes were observed in response to RE. The authors hypothesized that this EE-sensitive change may resemble a disturbance to the gut microbiome in response to a novel stimulus (i.e., EE), followed by a recovery after acclimation to the exercise stimulus (116). Smith et al. (2022) also investigated RE alone, but contrary to the previous study, they reported that a 10-week whole-body RE regimen in healthy, young adults increases gut microbiome diversity, but not richness (117). Lastly, in the only study (to date) that has utilized a longitudinal design in elderly individuals, there were only minimal changes to gut microbial diversity and composition in Japanese men (age 62–76 years) with a 5-week EE intervention (118).

The literature available in the realm of exercise and the gut microbiome, particularly with aging, is scant. Taken together, these findings, while diverse, suggest that exercise may alter the host gut microbiome composition, diversity, and/or function, but the degree to which these modifications can be observed depend on the exercise intervention (modality, intensity, and duration), host species and characteristics (sex, BMI, and age), as well as study design (cross-sectional vs. longitudinal). Therefore, we challenge investigators to focus on longitudinal study designs that control for potential confounding variables and incorporate ages from across the lifespan, especially the elderly.

## Conclusions

The gut microbiome has drawn substantial attention over the past decade for the role it plays in host health and disease. While gut microbes have been studied for years, our understanding of the symbiotic relationship that the gut microbiome maintains with its host and the specific functions it performs continues to expand exponentially. The gut microbiome develops early in life and remains relatively stable through adulthood. The literature suggests that age-related alterations in the gut microbiome do exist, but the lack of well-controlled longitudinal studies make it difficult to discern whether differences in the composition of the gut microbiome are the result or cause of various age-related conditions observed in both diseased and healthy elderly populations. Future studies exploring aging should focus on functional characteristics of the microbial community, in addition to composition, as the host ages. Multi-omic approaches that assess not only the gut bacterial genome, but also the transcriptome, proteome, and metabolome, can provide a more comprehensive view of the gut microbial community and its role in maintaining host health.

Many different factors can alter the diversity, richness, composition, and function of the gut microbiota. In this review, we have discussed the potential effects of diet and exercise on the gut microbiota. The influence of diet and/or nutrition on the gut microbiome has been moderately investigated. The majority of studies point to healthy, well-balanced diets, rich in fiber, favor a diverse and well-functioning gut microbiome; while diets containing processed foods, high in sugar and fats, lean towards dysbiosis. Conversely, the influence of exercise on the gut microbiome is not quite as clear. While some rodent studies have reported a positive impact of exercise on the composition and function of the gut microbiota, human studies have provided conflicting results. In general, findings from the human studies suggest that fitness level and body composition, as well as diet/nutrition, can influence the degree to which the gut microbiota is altered with exercise. Nonetheless, there is not yet enough evidence to conclusively determine the effects of exercise on altering the gut microbiota, but a multi-omics analytic approach is warranted.

Taken together, there is some evidence that diet and exercise can influence the gut microbiota; however, the number of studies investigating these lifestyle factors, in combination with aging, are quite limited. Therefore, more longitudinal studies that control for confounding variables are desperately needed to gain a more comprehensive understanding of the influences that aging, diet, and exercise may impact the gut microbiota.

## Author contributions

CB, RB, and KZ: all contributed to the conception of this work. CB, MW, OA, RB, and KZ: all contributed significantly to the drafting of this work. CB, RB, and KZ: contributed to the revision of this work. All authors contributed to the article and approved the submitted version.
